# Sex, Age, and Skull Morphology Effects on Eye Size and Refractive Errors in Reindeer

**DOI:** 10.1002/ece3.74530

**Published:** 2026-11-01

**Authors:** Solveig Arnegard, Andreas Zedrosser, Christine Wildsoet, Rigmor C. Baraas

**Affiliations:** ^1^ National Centre for Optics, Vision and Eye Care, Faculty of Health and Social Sciences University of South‐Eastern Norway Kongsberg Norway; ^2^ Department of Natural Sciences and Environmental Health University of South‐Eastern Norway Bø Norway; ^3^ Institute of Wildlife Biology and Game Management University for Natural Resources and Life Sciences Vienna Austria; ^4^ Herbert Wertheim School of Optometry and Vision Science University of California Berkeley California USA

**Keywords:** eye size, ocular dimensions, *Rangifer tarandus tarandus* (reindeer), refractive error, skull morphology, vision‐related sexual dimorphism

## Abstract

Eye size influences captured light and visual resolution and is used as an indicator of the relative importance of vision. Although vision‐related sexual dimorphism is subtle among vertebrates, Cervids exhibit pronounced sexual dimorphism in body size, antler development, and reproductive strategies. We tested the hypothesis that male reindeer ( *Rangifer tarandus tarandus* ) with larger skeletal dimensions would have larger eyes and narrower binocular overlap but similar refractive errors compared to females. We collected skull morphological, ocular biometric, and refractive error data from 69 semi‐domesticated reindeer (0.5–12.5‐year‐old; 27 females, 42 males) living year‐round under extreme high‐latitude seasonal variation in photoperiod in south‐central Norway. The relationships between eye size, mandible length, interorbital distance, and orbital diameter were analyzed, and binocular overlap and anterior blind zones were calculated. Eye size increased with measures of skull size, while refractive error remained minimal across all ages. Although male calves had significantly larger mandibles and interorbital distances, their orbital diameters were the same as in female calves. However, in adults, males also had larger orbital diameters. After controlling for differences in skeletal measures, males were found to have relatively smaller eyes than females, whereas binocular overlap and blind zones were similar. These findings demonstrate vision‐related sexual dimorphism in reindeer.

## Graphical abstract

We tested the hypothesis that male reindeer ( *Rangifer tarandus tarandus* ) with larger skeletal dimensions, would have larger eyes and narrower binocular overlap, but similar refractive errors compared to females. After controlling for differences in skeletal measures, males were found to have relatively smaller eyes than females, whereas binocular overlap and blind zones were similar. These findings demonstrate vision‐related sexual dimorphism in reindeer.

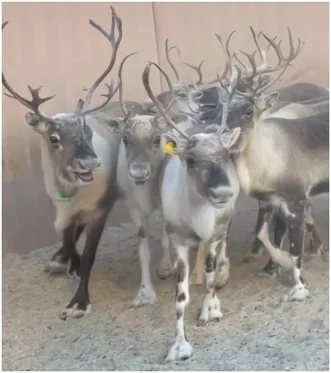

## 1 Introduction

Vision plays a fundamental role in mammalian survival and reproduction, supporting key behaviors such as foraging, predator avoidance, communication, inter‐ and intraspecific competition, mate recognition, offspring care, as well as decisions related to habitat selection and movement (Dimitrova and Merilaita 2009; Horth 2007; Tisdale and Fernández‐Juricic 2009). Because these behaviors are tightly linked to fitness, the mammalian visual system has been shaped by strong selective pressures to perform optimally within each species' ecological niche (Hughes 1977). Species inhabiting low‐light environments face unique challenges, with competing needs of a highly sensitive visual system yet with sufficient spatial resolution to support functional vision and ecologically relevant tasks (Cobo et al. 2025). Such trade‐offs highlight how ecological conditions drive the evolution of sensory systems, producing visual specializations matched to a species' behavioral and environmental demands.

Eye size is often used as an indicator of visual ability, due to its potential to influence both light capture and visual spatial resolution (Howland et al. 2004; Hughes 1977; Land and Nilsson 2012; Walls 1942). Eye growth, the ultimate determinant of eye size, is a key aspect of mammalian visual development (Hughes 1977). However, the evolutionary mechanisms regulating eye growth to ensure emmetropization, that is, a match between eye size and its optical components for clear (in‐focus) distance vision, can become dysregulated, thereby resulting in refractive errors. Eye growth is thought to be controlled via a negative feedback system with a set‐point (e.g., Wallman and Winawer 2004), which can be regarded as a predetermined degree of refractive error established by adolescence. For example, an eye that is too long for its optical power results in myopia (near‐sightedness), while an eye that is too short leads to hyperopia (far‐sightedness) (Zadnik et al. 2003). At birth, mammals typically show hyperopia, which slowly declines as their eyes grow and develop, eventually resulting in emmetropia (no refractive error) when adult (Flitcroft 2014; Howlett and McFadden 2007; Norton and McBrien 1992; Qiao‐Grider et al. 2007; Troilo et al. 2019). Mammals living outdoors in their natural ecological environments may be less likely to exhibit accelerated eye growth (too much growth), insofar as daylight exposure may support eye‐growth regulation, although dysregulated eye growth is known to involve both genetic and environmental influences. However, empirical support for this hypothesis is limited. Most studies on mammalian eye growth have used laboratory animal housed under artificial light conditions (e.g., Troilo et al. 2019), while studies of wild species (e.g., white‐tailed deer; *Odocoileus virginianus* ) typically involve very small sample sizes (median *n* = 4) (D'Angelo et al. 2008; Gur and Sivak 1979; Howland et al. 1993; Murphy et al. 1992; Ofri et al. 2004; Sivak et al. 1989).

Binocular overlap, that is, the portion of the visual field that is seen by both eyes simultaneously, influences the total field of view, as well as depth perception (Walls 1942). Patterns of binocular overlap generally reflect adaptation to a mammal's role as predator or prey (Heesy 2004; Hughes 1977; Land and Nilsson 2012; Walls 1942). Predators typically have forward‐facing eyes with significant binocular overlap and a comparatively small total field of view. In comparison, prey species tend to have laterally positioned eyes, with little binocular overlap, yet a wide field of view (Heesy 2004; Hughes 1977; Land and Nilsson 2012; Walls 1942). Even a small degree of binocular overlap, however, may provide a substantial visual advantage, by enabling some level of binocular depth perception, thereby helping prey species to spot predators and navigate their environment while moving at speed (Read 2021).

In mammals, eye growth and size, the extent of binocular overlap and the incidence of refractive errors may exhibit sexual dimorphism, likely reflecting inter‐sexual differences in life‐history traits, mating strategies, or foraging/hunting strategies (Clutton‐Brock and Vincent 1991; Ruckstuhl and Neuhaus 2000; Trivers 1972). Humans display sexual dimorphism in eye size, with males having larger eyes than females for a given refractive error (Hagen et al. 2019; Tideman et al. 2018). This difference likely reflects differences in overall body size, with human males being generally taller than females (Rodriguez‐Martinez et al. 2020). Nonetheless, vision‐related sexual dimorphism in vertebrates is generally reported to be subtle and not associated with structural differences, in contrast to findings in insects (Cronin et al. 2014).

Here we use the European Mountain reindeer ( *Rangifer tarandus tarandus* ), a prey species with laterally positioned eyes (Newman and D'Angelo 2024) and pronounced sexual dimorphism (Bowyer 2004; Holand et al. 2004; Lincoln 1992; Morrison and Whitridge 1997), as model species to investigate possible sexual dimorphism in eye size, refractive error, and binocular overlap in mammals. The selected reindeer model represents a mammal living under natural light conditions year‐round. In Europe, they live at northern latitudes (above ~58° N) where natural light conditions range from near‐total darkness during winter (< 6 h of daylight) to near‐continuous sunlight in summer (> 19 h of daylight). We measured the skulls and eyes of 69 male and female free‐ranging semi‐domestic reindeer spanning a wide range of ages. Mandible length was used as a proxy for body size (Skogland 1983). We hypothesized that (1) males have larger mandible length, interorbital distance and orbital diameter than females; (2) the length of the eye (i.e., axial length) and of its other components, such as the vitreous chamber and crystalline lens, increase with age, with males having larger eyes compared to females, but not necessarily differing in refractive error; (3) compared to females, males have narrower binocular overlap, measured as width of the anterior blind zone.

## 2 Methods

The heads and eyes of 69 semi‐domestic European Mountain reindeer were collected in collaboration with Filefjell Reinlag (Filefjell Reindeerherder Association; https://www.filefjell‐reinlag.no/), the southern‐most reindeer husbandry unit in Norway. These reindeer are part of a managed semi‐domestic herd that lives year‐round in the wild in an area spanning ~60°20′–61°20′ N; 8°20′–9°50′ E. Herders guide the herd from their summer range in the north‐western part of the area to the winter range in the southeast, following an annual rhythm that aligns with the changing seasons (Pape and Löffler 2015). As part of herd management, select animals are slaughtered each winter, in a mobile slaughter facility over a two‐week period in November–December. Apart from the guided migration and slaughter period, reindeer roam unrestricted under the condition of daily boundary surveillance, whereby the owners monitor the herds via GPS‐collars attached to some older female animals, to ensure that they stay within the permitted area and do not intermix with wild reindeer in adjacent areas (Pape and Löffler 2015). The pre‐calving herd size has been stable at ~3000 individuals for the last 10 years, with the sex‐age composition kept at 78% adult females, 1% adult males, and 21% calves (0–1 year‐old) (Direktoratet for forvaltning og økonomistyring [The Norwegian Agency for Public and Financial Management] 2024).

**FIGURE 1:**
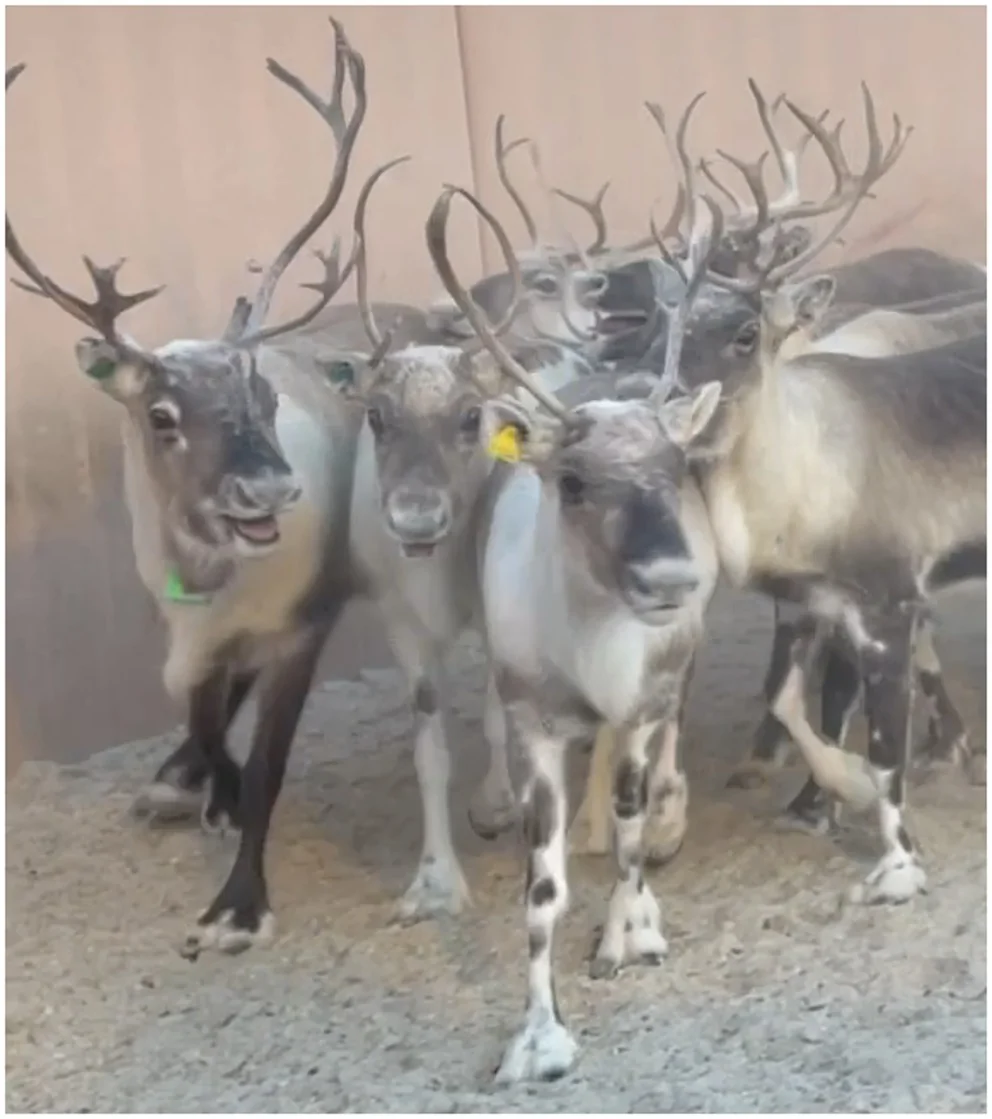
Semi‐domesticated European mountain reindeer ( *Rangifer tarandus tarandus* ) included in the study, gathered in an enclosure prior to slaughter selection, illustrating the typical appearance of the animals. The photograph was taken by RCB in November 2023 of a herd owned by Filefjell Reinlag at Filefjell, Norway.

All samples were collected and measured between 26 November and 1 December 2023, corresponding to the annual slaughter (Figure 1). Specifically, heads were collected from randomly selected male and female calves (0.5‐year‐old (y.o.)), which was the only age group where a direct comparison of ocular parameters of the two sexes was possible. Additional samples were collected from 1.5 and 2.5‐y.o. males (no females in these age groups were slaughtered), as well as 10.5 and 12.5‐y.o. females (no males within these age groups were slaughtered). As all calves are ear‐tagged annually, ear tags from individual animals were collected to determine their birth year and obtain age information. All reindeer ≥ 1.5 years were considered adults (Weladji et al. 2002).

For slaughter, reindeer were stunned with a bolt gun to the brain and their heads removed, usually within a minute after stunning. The heads were usually obtained within 5 min of slaughter and immediately transferred to a mobile laboratory, where they were positioned on a custom‐designed stand, oriented to simulate head posture during walking, that is, raised head (Appendix S1). Ocular refractive error and biometric parameters, as summarized below, were obtained from both right and left eyes.

Refractive errors were measured by a trained optometrist using a hand‐held streak retinoscope (Keeler Professional, Keeler Ltd., Windsor, Berkshire, England), combined with a lens rack (+1.00–+10.00 D, +/−0.50 D steps, Rodenstock GMBH—opti, Munich, Germany). Retinoscopy is a standard clinical technique used to measure refractive errors, both in humans (American Academy of Ophthalmology 2024) and in animals with single lens eyes, like humans (Davidson 1997). Eyes were not cyclopleged, based on the assumption that the ciliary muscle and thus accommodation would be relaxed post‐mortem, as reported in both humans (Saukko and Knight 2015) and other animals (Bueno et al. 2017). Likewise, the ocular pupils were found to be fully dilated. The eyelids also remained in their natural open position post‐mortem, allowing for direct examination and measurement of the eyes. Refractive error measurements were made along both the horizontal (180°) and vertical (90°) meridians. The refractive error results for each meridian, as measured in dioptres (D), were then used to calculate the spherical equivalent refractive error (SER), defined as SER = sphere +½ cylinder. The sphere was determined by the most positive meridian of retinoscopy, while the cylinder, representing the astigmatic error (DC, expressed dioptres), was calculated as the difference in the results for the two meridians. Refractive errors were subsequently classified as either myopic (SER ≤ −0.50 D), emmetropic (−0.50 D < SER < + 0.50 D), or hyperopic (SER ≥ + 0.50 D). Eyes exhibiting meridional differences ≥ 1.00 D were classified as having astigmatism. To assess the accuracy and repeatability of the retinoscopy measurements, three randomly selected animals were independently examined by a second trained optometrists and the two sets of results compared.

Axial ocular dimensions were measured in situ using A‐scan ultrasonography (Accutome A‐Scan Plus Connect, Accutome Inc., Malvern, PA, USA); axial length (AXL), anterior chamber depth (ACD), and lens thickness (LT) were thus measured directly, with vitreous chamber depth (VCD) subsequently calculated by subtracting ACD and LT from AXL. The instrument's accuracy was 0.1 mm, as specified by the manufacturer for the 10 MHz A‐Scan probe used for these measurements (Keeler 2024). AXLs measured with Accutome A‐Scan have also been reported to be highly correlated with AXL data collected from the same eyes with partial coherence interferometry (PCI) devices, such as the Zeiss IOLMaster 500 (Srivannaboon et al. 2013). In preparation for and during ultrasonography measurements, the corneas of eyes being measured were moistened with physiological saline, thereby ensuring good contact between the probe and the anterior corneal surface. The amplitude of the waveforms was then adjusted as needed to ensure signal acquisition (range: 67–100 dB), and five measurements were captured using the instrument's auto mode. These data were used to derive for each eye, means (x̄) and standard deviations (SD) for ACD, LT, and AXL. We excluded individual measurements that deviated more than one SD from x̄, thereafter recalculating means based on the remaining values for use in further analysis. In the case of lens thickness, these data were compared with additional measurements made on a subset of isolated lenses after enucleation and dissection, using a digital caliper with ceramic tipped jaws (Insize Europe S.L, Derio, Spain).

After enucleation of both eyes, the skulls were skinned and gently boiled to remove meat and reveal the skull structures from which vision‐related landmarks were evaluated. The orbital diameters were measured along four axes: horizontal, vertical, 45°, and 135°, using the bone “fine structure” around the orbit as landmarks to ensure that measurements were always carried out the same way (Appendix S2, panel D). Mean orbital diameters were calculated as the average of these four measurements. An orbital axis angle (*α*) was also estimated based on the orientation of the horizontal orbital axis. Finally, the length of the left mandible was measured as described by Blant and Gaillard (2004), and used as a proxy for overall body size (Pérez‐Barbería et al. 2014; Skogland 1983) (Appendix S2, panel A). Three measurements were made and averaged to calculate mean individual mandible length. Because we only measured left mandibles, we also limit ocular data presentation to the left eyes, except in the case of interocular comparisons. Two measurements of the interorbital distance were made, corresponding to the most anterior (interorbital anterior distance, IOAD) and most posterior (interorbital posterior distance, IOPD) aspects of the orbital rim (Appendix S2, panels B and C). These measurements were obtained with a digital caliper (Digital Caliper Limit CDN 300, Limit, Sweden) and repeated twice, with the mean used in further analyses.

**FIGURE 2:**
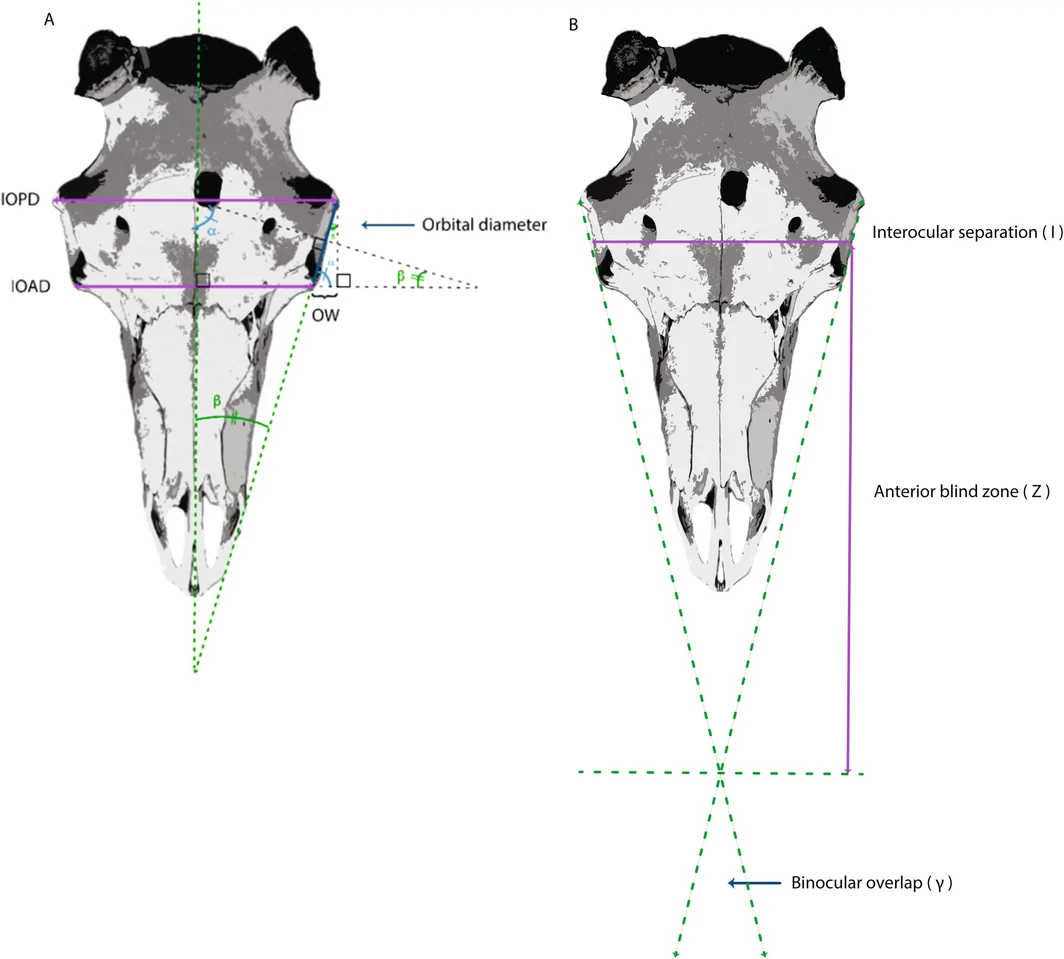
Illustration of the parameters measured from skulls of semi‐domestic reindeer collected in south‐central Norway, December 2023: An overview of obtained skull measurements interorbital anterior distance (IOAD), interorbital posterior distance (IOPD), orbit width (OW), and horizontal orbital diameter, and the estimates of orbital axis angle (*α*) and orbital convergence (*β*) (A), and binocular overlap (*γ*) and anterior blind zone (*Z*), where *I* is the interocular separation between the two centres of projection (B).

The interorbital anterior distance (IOAD), interorbital posterior distance (IOPD), and horizontal orbital diameter were used to estimate the orbital axis angle and binocular overlap (Figure 2) as described in Mitchell et al. (2013). The estimated orbital axis angle (*α*) was derived from the ratio of the orbit width (OW), representing the difference between IOAD and IOPD, and the horizontal orbit diameter (Figure 2A). The orbital axis angle, which represents the cosine of the latter ratio, was then used to estimate the binocular overlap (*γ*) based on the following equation:

$$\gamma =2*\left(90-\text{orbital axis angle}\right)$$

The orbital axis angle data were also used to estimate orbital convergence (*β*), calculated as follows: 90°–orbital axis angle. This method of estimating orbital convergence was validated by comparison with a second set of measurements derived for a subset of 14 skulls, which were imaged in the laboratory using raw format capture (iPhone 15 Pro Max, Apple Inc). Orbital convergence was measured from these images using ImageJ (Schneider et al. 2012). There was no significant difference in the orbital convergence angle estimates obtained using these two methods (paired *t*‐test; *t* = 0.245, *p* = 0.788).

The anterior binocular blind zone (*Z*) (i.e., the region without visual input immediately in front of the nose of the animal, between its eyes and the start of the binocular overlap, Figure 2B) was also estimated for individual skulls using the formula from Read (2021),

$$Z=\frac{0.5I}{\tan\left(0.5\gamma \right)}∼\frac{I}{\gamma }$$

where *I* is the interocular separation between the two centres of projection, that is, the distance between the eyes, calculated as the mean of interorbital anterior and posterior distances (IOAD & IOPD), and *γ* represents the estimated binocular overlap, calculated from the orbital axis angle (*α*) (Figure 2B).

All data collection was carried out with approval from the Norwegian Food Safety Authority (FOTS, ID 30352) and in accordance with the ARVO Statement for the Use of Animals in Ophthalmic and Vision Research.

### 2.1 Statistical Analyses

We used parametric tests (paired *t*‐test, two‐sample *t*‐test, correlation coefficient) for descriptive statistics. To investigate sexual dimorphism, we used linear regression models with derived eye and vision parameters (axial length, SER, anterior blind zone) as response variables, and skeletal (mandible length, interorbital distance, orbital diameter) as well as eye parameters (axial length) as predictor variables. For each response‐predictor pair, four hierarchical linear models were constructed: (0) a null model without predictors (*y*~1); (1) a model with a single skeletal or eye parameter as predictor; (2) a model including the predictor, as well as sex (as factor variable, with female = 0, male = 1); (3) a model including the predictor, sex, and the interaction term predictor*sex. We investigated the following relationships: axial length ~ mandible length; axial length ~ interorbital distance; axial length ~ orbital diameter; SER ~ axial length; anterior blind zone ~ mandible length. These models were then compared using Analysis of Variance (ANOVA). Model #1 was significantly better than the null model for all relationships and the final model was selected based on the principle of parsimony, retaining predictors only when they significantly improved model fit (Appendix S3). Improvements were evaluated using changes in *R* ^2^ and tested for significance with F‐statistics and their associated *p*‐values. See Appendix S3 for detailed method description and Table C1, which summarizes the results for all models tested. Additionally, hierarchical multiple linear regression analyses were conducted with AXL as the dependent variable and LT, VCD, and ACD as predictors. A *p*‐value of ≤ 0.05 was considered statistically significant, and a *p*‐value 0.05 < *p* < 0.1 was considered a statistical trend (Zuur et al. 2007). All analyses were carried out in R‐Studio, version 2024.09 (R Core Team 2024). All supporting information are available in the DataverseNO repository https://dataverse.no/previewurl.xhtml?token=6ca93bcd‐4ae4‐4d7c‐bc4b‐daabce894e5e.

## 3 Results

The composition of the samples evaluated (Table 1) reflected the reindeer owners' selection of ~2000 healthy animals for slaughter, grouped according to age (calves and adults ≥ 1.5‐y.o). The sample included 0.5‐y.o. calves (*n* = 35, females = 17, males = 18), 1.5‐y.o. males (*n* = 16), 2.5‐y.o. males (*n* = 8), 10.5‐y.o. females (*n* = 9), and 12.5‐y.o. females (*n* = 1).

### 3.1 Skull Morphology

Male calves had significantly longer mandibles and wider interorbital distances compared to female calves (*t*‐test, *t* = −3.373, *p* = 0.002 and *t*‐test, *t* = −4.332, *p* < 0.001, respectively; see Table 1 for mean and median measurements). There was no sex‐related difference in the orbital diameters of the calves (left eyes: *t*‐test, *t* = −0.452, *p* = 0.654). Mandible length, interorbital distances, and orbital diameter increased with age (Figure 3A–C, and Table 1) (Additional data in Appendix S4). Although the data do not allow for direct comparisons of adults of different ages, comparison of 2.5‐year‐old males and 10.5‐year‐old females, representing the oldest age classes of each sex in our sample, with females having attained full skeletal size, revealed a significantly greater interorbital distance in males (*d* = 2.33, *p* < 0.001; Figure 3B). Mandible length also tended to be greater in males (*d* = 1.07, *p* = 0.060; Figure 3A). No significant sex difference was detected for orbital diameter (*d* = 0.69, *p* = 0.170; Figure 3C).

**FIGURE 3:**
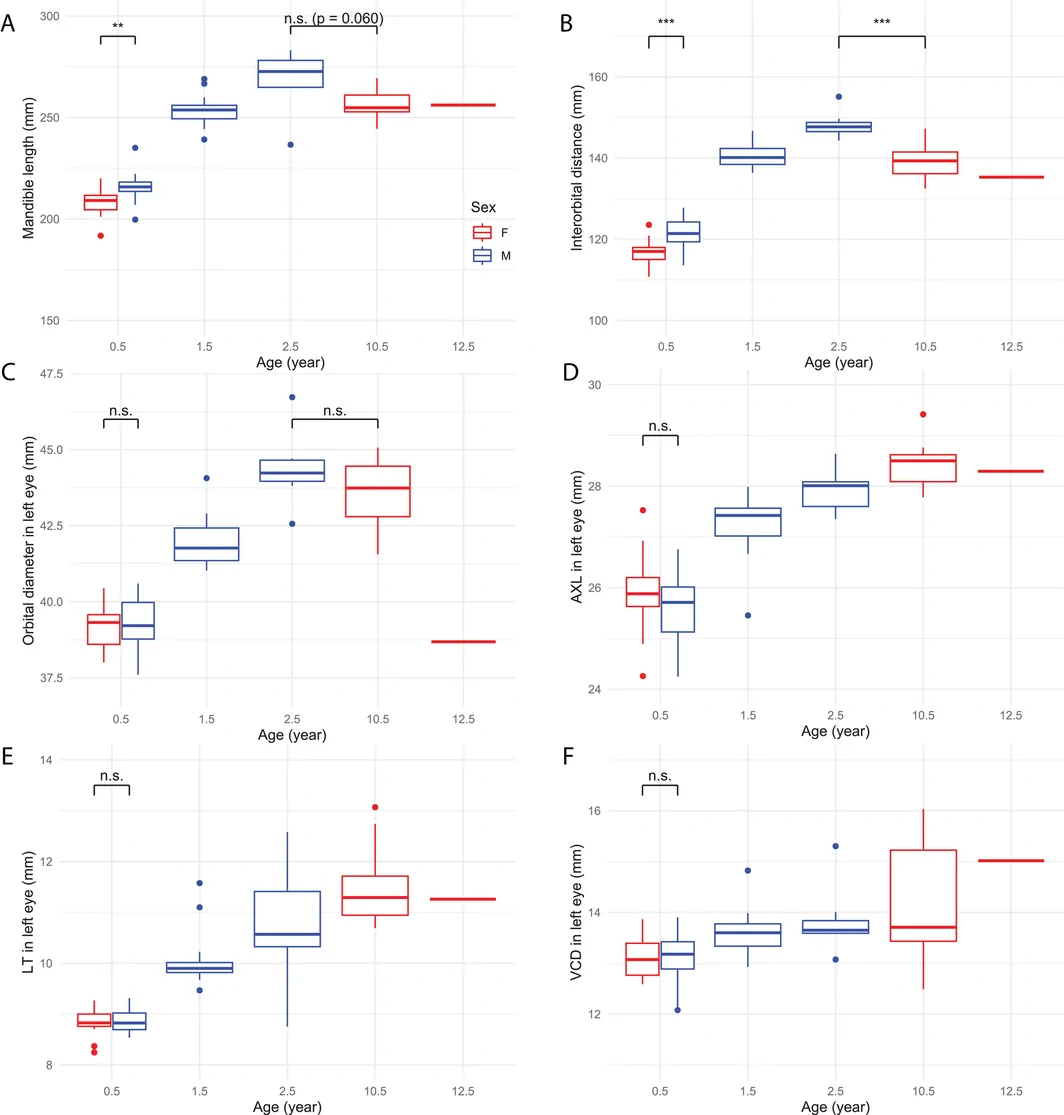
Skeletal and ocular parameters, (A) Mandible length, (B) Interorbital distance, (C) Orbital diameter, (D) Axial length (AXL), (E) Lens thickness (LT), (F) Vitreous chamber depth (VCD), as a function of age for female (red) and male (blue) semi‐domestic reindeer collected in South‐Central Norway, December 2023. Statistical significance is indicated as follows: N.s., not significant (*p* ≥ 0.05); **, *p* < 0.01; ***, *p* < 0.001.

**TABLE 1:** Descriptive statistics (mean, median, and range) for left eye axial length (AXL), mandible length, interorbital anterior distance (IOAD), interorbital posterior distance (IOPD), and orbital diameter (Orb. ⌀) (left side only), grouped according to developmental stage (calves and adults), of semi‐domestic reindeer (female and male) collected in South‐Central Norway, December 2023 (*n* = 69).

	Calves				Adults			
	*n*	Mean	Median	Range	*n*	Mean	Median	Range
AXL (mm)								
All	35	25.7	25.8	24.2–27.5	34	27.7	27.8	25.5–29.4
F	17	25.9	25.9	24.3–27.5	10	28.4	28.4	27.8–29.4
M	18	25.6	25.7	24.2–26.8	24	27.4	27.5	25.5–28.6
Mandible length (mm)								
All	35	212.2	212.9	191.7–235.0	34	258.0	255.7	236.6–283.1
F	17	208.2	209.1	191.7–219.9	10	256.2	255.5	244.4–269.3
M	18	216.0	215.8	199.7–235.0	24	258.7	255.7	236.6–283.1
IOAD (mm)								
All	35	108.8	109.1	97.7–118.0	34	130.8	130.7	120.5–143.5
F	17	106.6	106.8	97.7–114.1	10	127.7	127.7	120.5–134.7
M	18	110.9	110.9	103.3–118.0	24	132.1	130.9	124.6–143.5
IOPD (mm)								
All	35	129.8	129.2	122.2–138.5	34	152.6	152.4	143.6–166.8
F	17	126.9	127.0	122.2–133.0	14	149.2	148.7	143.6–160.6
M	18	132.6	132.3	124.0–138.5	24	154.0	153.6	145.6–166.8
Orb. ⌀ (mm)								
All	35	39.3	39.3	37.6–40.6	34	42.9	42.7	38.7–46.7
F	17	39.2	39.3	38.0–40.4	10	43.1	43.3	38.7–45.1
M	18	39.3	39.2	37.6–40.6	24	42.8	42.5	41.0–46.7

### 3.2 Ocular Dimensions

Axial length (AXL), lens thickness (LT), and vitreous chamber depth (VCD) increased with age (Figure 3D–F), while anterior chamber depth (ACD) decreased with age (data not shown). There was no difference in axial length between either female and male calves (*t*‐test, *t* = 1.16, *p* = 0.253) or female and male adults (*t*‐test, *t* = 0.56, *p* = 0.581). Mean, median, and range axial length (AXL) data are also summarized in Table 1. In a hierarchical multiple linear regression model with AXL as the dependent variable, LT explained 62% of the variability in AXL (*R* ^2^ = 0.616, *p* < 0.001, Figure 4D). ACD and VCD explained an additional 25% and 14% (*R* ^2^ change = 0.248 and 0.136, respectively, both *p* < 0.001).

AXL increased with increasing mandible length (Model #1: *β* = 0.811, *p* < 0.001; Figure 4A), interorbital distance (Model #1: *β* = 0.765, *p* < 0.001; Figure 4B), and orbital diameter (Model #1: *β* = −0.794, *p* < 0.014; Figure 4C). Nonetheless, in the model controlling for sex‐related differences in skull morphology (which were enlarged in males), males were found to have significantly shorter AXLs compared to females relative to mandible length (*β* = −0.312, *p* < 0.001), interorbital distance (*β* = −0.396, *p* < 0.001), and orbital diameter (*β* = −0.183, *p* < 0.001). These models (Model #2) provided a significantly better fit to the data than Model #1 (summaries of the linear regression models and ANOVA model comparisons are presented in Appendix S3).

**FIGURE 4:**
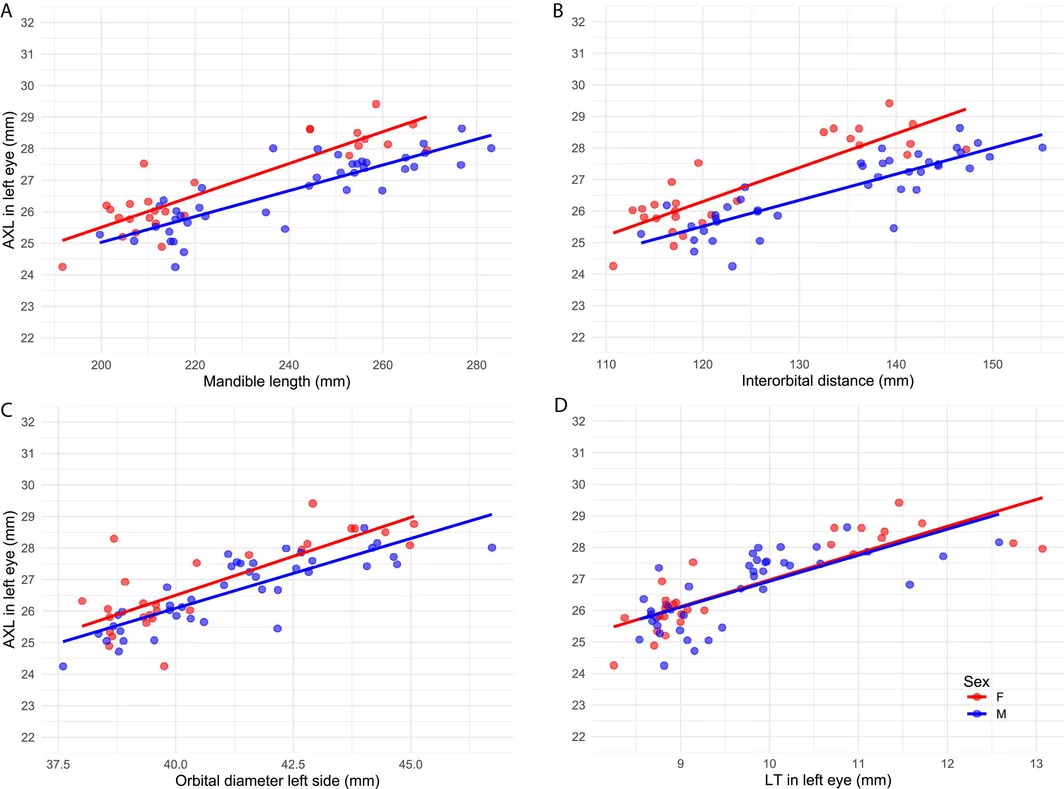
Axial length (AXL) in left eye, as a function of (A) Mandible length, (B) Interorbital distance, (C) Orbital diameter, and D: Lens thickness (LT), categorized by sex (female, F = red; male, M = blue) and with a trendline fitted by linear regression, of semi‐domestic reindeer collected in South‐Central Norway, December 2023.

### 3.3 Refractive Errors

The overall median SER was 1.00 D, with the majority of the reindeers (82%, *n* = 54) exhibiting mild hyperopia (≥ + 0.50 D SER ≤ + 2.00 D), and only a small number (18%; *n* = 12) exhibiting emmetropia (−0.50 D < SER < + 0.50 D), and none exhibiting myopia (≤ −0.50 D) (Table 2). The SERs of the right and left eyes of individual animals were also highly correlated (*r* = 0.730, *p* < 0.001). Astigmatism (≥ 1.00 DC) was observed in 13% (*n* = 9), out of which seven had with‐the‐rule astigmatism (greater refractive power along the vertical meridian). There was no significant sex‐related difference in SER, as reflected in the data from female and male calves (left eye data, *t* = −1.98, *p* = 0.057), and female and male adults (*t* = −1.69, *p* = 0.097), although males tended to be slightly more hyperopic than females. None of the animals had significant anisometropia, that is, refractive error difference ≥ 1.00 D between their two eyes. SERs decreased significantly with increasing axial length for both sexes (*β* = −0.363, *p* = 0.003). However, neither the effect of sex nor its interaction with SER was significant. Details of data analyses are available in the supporting information (see Appendix S3).

**TABLE 2:** Profile of the sample, based on age (calves = 0.5‐y.o.; adults ≥ 1.5‐y.o.), sex (F = female, M = male), sample size (*n*), along with refractive errors expressed in terms of spherical equivalent refractive error (SER, D) and astigmatism (cylinder, DC] for the left eyes of semi‐domestic reindeer collected in South‐Central Norway, December 2023.

Age	Sex	*n*	SER [D]		Cylinder [DC]	
			Median	Range	Median	Range
Calves						
	Overall	35	1.00	0.25–1.50	−0.25	−1.50–0.00
0.5	F	17	0.75	0.25–1.50	−0.50	−1.50–0.00
0.5	M	18^a^	1.13	0.50–1.50	0.00	−0.50–0.00
Adults						
	Overall	34	0.75	−0.25–1.75	−0.50	−1.50–0.00
1.5	M	16^a^	1.19	0.00–1.75	−0.50	−1.00–0.00
2.5	M	8	0.69	−0.25–1.50	−0.00	−0.50–0.00
10.5	F	9	0.50	0.00–1.75	−0.50	−1.50–0.00
12.5	F	1	0.75	—	−1.50	—

^a^ Missing refractive data for one 0.5‐year‐old and two 1.5‐year‐old.

### 3.4 Interocular Comparisons and Potential for Binocular Depth Perception

There was a high correlation between AXL in the two eyes (females, *r* = 0.891; males, *r* = 0.778; Figure 5A) and between right and left orbital diameter (females, *r* = 0.963; males, *r* = 0.974; Figure 5B). There was no difference in absolute AXL differences (|Right eye—Left eye|, *t* = −0.344, *p* = 0.732) between sexes.

**FIGURE 5:**
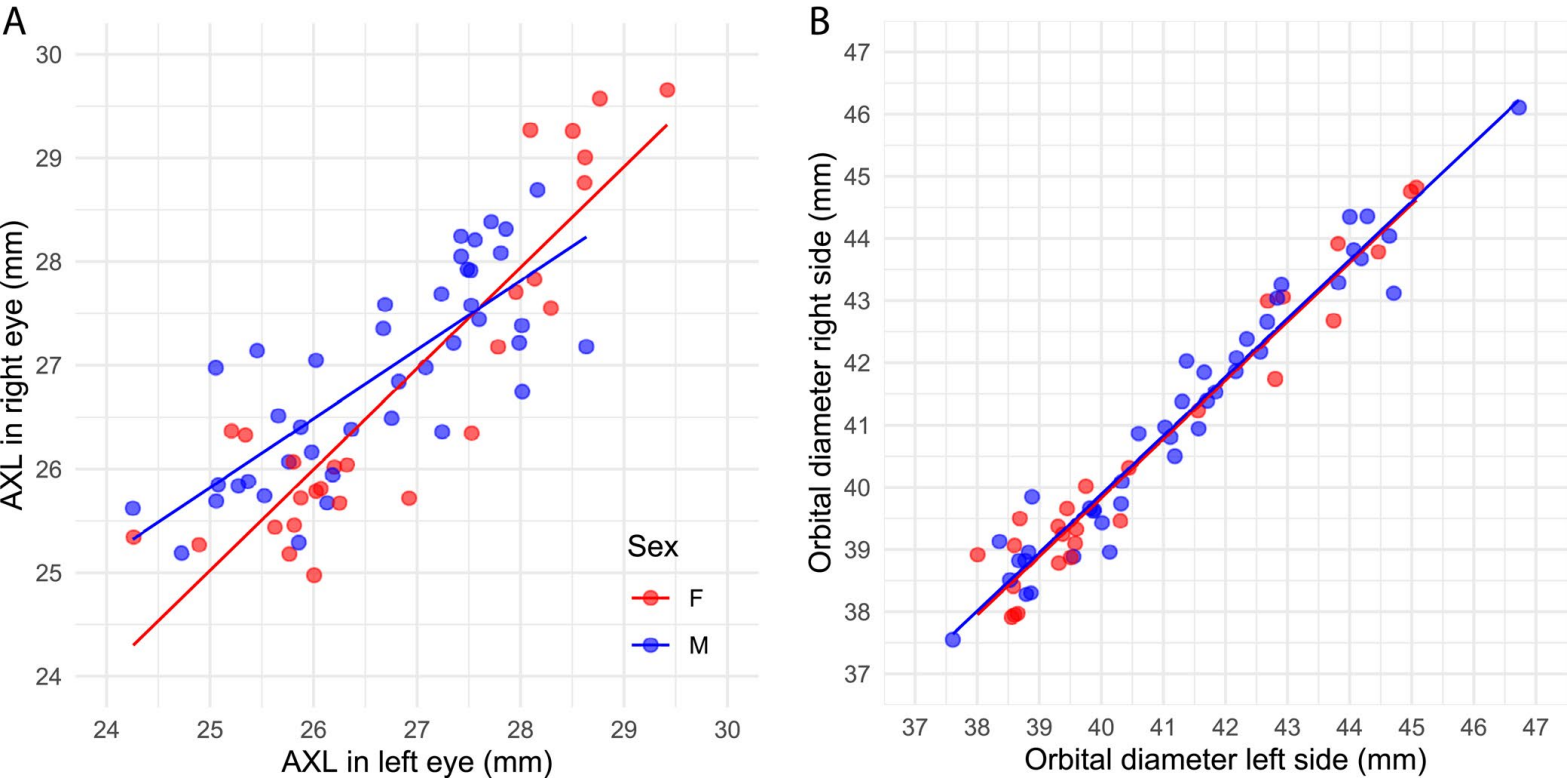
(A) Axial length (AXL) of right eyes plotted as a function of AXLs of left eyes, and (B) Orbital diameter on the left side of the skull as a function of orbital diameter on the right side, categorized by sex (female, F = red; male, M = blue) and with a trendline fitted by linear regression of semi‐domestic reindeer collected in South‐Central Norway, December 2023.

#### 3.4.1 Anterior Blind Zones

The estimated anterior blind zone increased with age (Figure 6A), and with increasing mandible length for both sexes (*β* = 0.553, *p* < 0.001, Figure 6B). The effect of sex was not significant, nor was its interaction with mandible length (Appendix S3).

**FIGURE 6:**
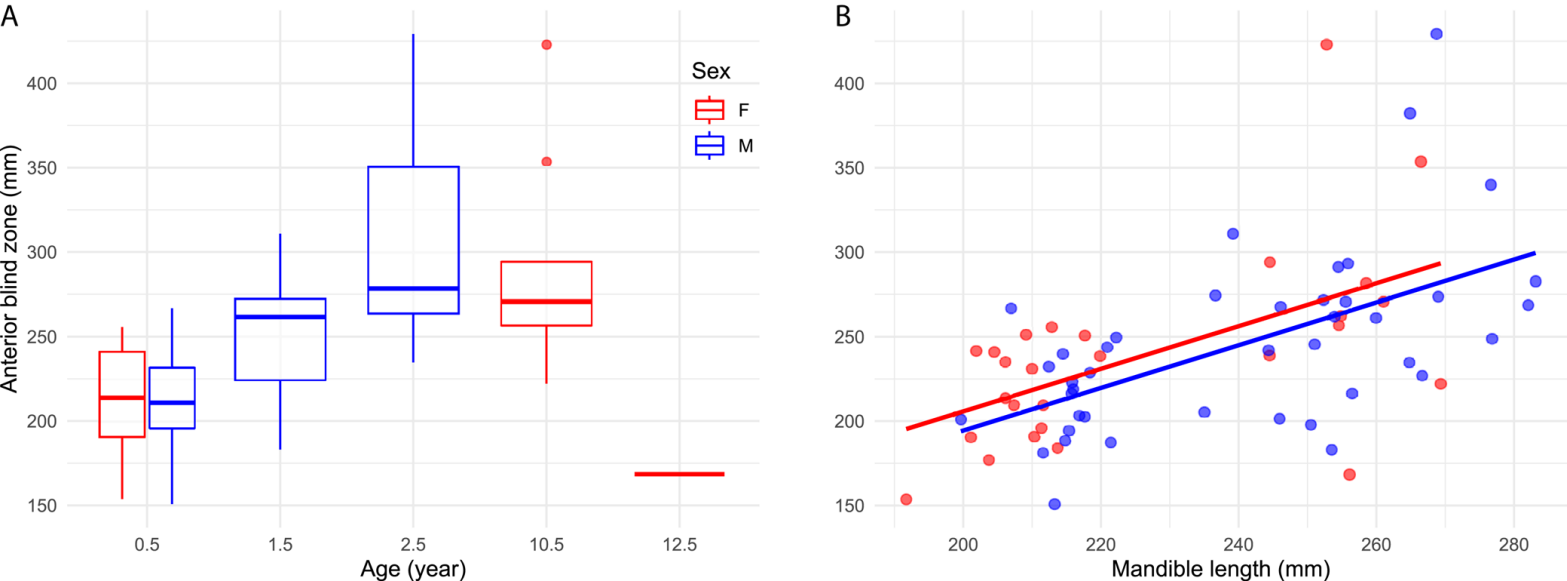
Boxplot showing the distribution of anterior blind zone estimates (mm) across age groups (in years), categorized by sex (female, F = red; male, M = blue) (A), and estimated anterior blind zone as a function of mandible length, categorized by sex (female, F = red; male, M = blue) and with a trendline fitted by linear regression (B) in semi‐domestic reindeer collected in South‐Central Norway, December 2023.

## 4 Discussion

We obtained skull morphology, ocular biometry, and objective measures of refractive errors from 69 male and female free‐ranging semi‐domestic reindeer aged 0.5–12.5 years. Male calves had significantly longer mandibles and greater interorbital distances than female calves, and adult males exhibited significantly greater interorbital distances and a trend towards longer mandibles. However, no sex differences were detected in orbital diameter in calves (partial support of hypothesis 1). There were no sex‐related differences in eye size, as reflected in the axial length data, although eye size increased with age. However, when controlling for skull morphology (mandible length, interorbital distance, and orbital diameter), male reindeer had relatively shorter axial lengths than females (partial support of hypothesis 2). We found no sex‐related differences in refractive error or binocular overlap. The absence of such differences opposes the prediction made in hypothesis 3.

### 4.1 Sexual Dimorphism in Skull Morphology and Eye Size

Male reindeer calves had larger skeletal and bone structures. Thus, the lack of sex‐related differences in AXLs and orbital diameters implies that female calves have relatively larger eyes. Male calves are generally larger at birth, and their faster growth and greater adult size align with the reproductive strategy of a polygynous system, in which male reproductive success depends on body size (Loison and Strand 2005). Because skeletal growth is an investment in future reproductive success, male reindeer prioritize growth, as dominance, size, and strength strongly influence their ability to compete for mates (Espmark 1964; Røed et al. 2002). The lack of difference in absolute measures of AXL in calves could therefore be due to sex‐related differences in energy allocation involving a trade‐off in males between the growth of eyes and the growth of the larger skull and antlers in favor of these non‐ocular structures to maximize reproductive success (Andersson 1994). Combined, these results tentatively suggest the existence of vision‐related sexual dimorphism in Cervids. This interpretation is supported both by the direct comparisons available in calves and by the patterns observed in older age groups, where females have attained full skeletal size, while the available data indicate that males may continue to grow. Accordingly, the pooled models provide a sensible approach for examining sexual differences, even though they cannot be regarded as definitive tests of adult sexual dimorphism in the absence of age‐matched adult males and females.

Interocular differences in either ocular axial length and/or in the eye's optical components can lead to differences in the refractive errors of the two eyes of an individual. This may result in impaired binocular vision, and thereby the ability to judge distances of nearby objects (Acar et al. 2019). While we observed stronger symmetry in AXL between the two eyes in females than in males (Figure 5A), none of the animals showed significant interocular differences in their refractive errors, which were also relatively small, especially in older animals. It is our conjecture that for females, their greater ocular symmetry may benefit binocular vision, allowing for quick detection of threats in the environment without extensive head movements (Heesy 2008; Holden and Low 1989; Read 2021; Walls 1942).

The finding that eye size in itself is similar between male and female reindeer contrasts with what is known about human eyes (e.g., Tideman et al. 2018), where sexual dimorphism in eye size is known to be present from infancy (Isenberg et al. 1995). Normal eye growth (enlargement) in humans is mainly accounted for by changes in VCD, and VCD is larger in males than females. This difference is present at birth and remains through to young adulthood (Hagen et al. 2019; Larsen 1971). In reindeer, eye growth is mainly accounted for by increases in LT and to a lesser degree, in VCD. There is also evidence of sexual dimorphism in the relative contribution of LT to AXL, with LT contributing more to age‐related changes in AXL in males than females. The relatively large contribution of lens thickness to axial length raises the possibility that lens morphology may be relevant to vision under low‐light winter conditions, but this hypothesis requires direct optical and functional testing.

### 4.2 Refractive Errors

Reindeer were found to maintain mild‐to‐no refractive error (mild hyperopia to emmetropia), irrespective of age and sex. These findings are intriguing, as they were obtained from animals that experience extreme high‐latitude seasonal variation in photoperiod. The refractive error pattern is consistent with that their eyes can emmetropize effectively, despite the extreme annual variability in the environmental light conditions. Note that differences in age profiles of male and female reindeer represented in the study sample could account for some observed sex‐related differences. Studies have shown that the body weight of semi‐domestic male reindeer increases until about five years of age (Melnycky et al. 2013; Nieminen and Petersson 1990). In the current study, the oldest males were 2.5 years old, while the adult females were 9–11 years older. Consequently, a further increase in skull dimensions and eye size up to this age in male reindeer is likely. However, in 2.5 years old males, the growth trajectory of skull dimensions remains steep, whereas that of eye size has plateaued (cf. Figure 3C,D). A similar slowing of eye growth is observed in other animals and in humans after the initial phase of emmetropization has been completed (c.f. figure 1 in Troilo et al. 2019). This suggests that the relative sex difference is expected to increase with age. That there is a greater contribution of LT to observed age‐related AXL changes and that males have relatively more hyperopic refractive errors warrants further investigation.

In contrast to what is observed in reindeer, the human eye exhibits regulated growth towards emmetropia in childhood and for those who maintain mild‐to‐no refractive error into adulthood, females tend to have significantly smaller eyes than males, with greater optical corneal power, as necessary to achieve and maintain emmetropia (Tideman et al. 2018; Flitcroft 2014). Consequently, this means that the eye growth regulation in humans has biologically adapted to sex‐related differences in eye size to achieve the same refractive error outcome. A weak correlation has been observed between a person's baseline height and the size of their eyes, but human eye growth appears to occur independently of increases in body height (Hagen et al. 2019; Nilsen et al. 2023). Interestingly, the data reported for reindeer similarly suggests that general skeletal growth occurs independently of eye growth.

While there is limited published refractive error data representing large wild mammals, some data are available for domesticated and laboratory animals. For example, domesticated ungulates, such as sheep ( *Ovis aries* ) and cattle ( *Bos taurus* ), were found to exhibit mild hyperopia (Bueno et al. 2017; Piggins and Phillips 1996), while horses ( *Equus caballus* ) are predominantly emmetropic with small refractive errors, though mild myopia or hyperopia and astigmatism occur (Bracun et al. 2014; Harman et al. 1999; Rull‐Cotrina et al. 2013). The origin of such refractive error differences remains unresolved, with plausible contributing factors including breed, trait selection, management practices (field or stable) (Bracun et al. 2014), and amount of exposure to artificial light.

Some insight into visual adaptations of wild ungulates is provided by a study of white‐tailed deer. The reported mean axial lengths of female fawns and adult deer were 25.3 and 27.8 mm respectively, with hyperopia of +7.96 D estimated for a 0.5‐year‐old animal based on a schematic eye with a multifocal lens (D'Angelo et al. 2008). This level of hyperopia significantly exceeds the hyperopic refractive errors observed in reindeer of a similar age in our study. However, noteworthy, white‐tailed deer and reindeer differ in their habitat preference, climate adaptations, environmental conditions, and social structures. White‐tailed deer live in more moderate climates compared to reindeer (Geist 1999); they are also a smaller, primarily forest‐dwelling species (McShea et al. 2012), generally living solitary lives (Hewitt 2011).

### 4.3 Binocular Vision

Irrespective of sex, the increased interorbital distance with increasing age and skull size results in a decrease of the binocular overlap and a larger anterior blind zone. This provides adult reindeer with a larger total visual field. This anatomical change likely enhances detection of predators, herd coordination, and navigation (Heesy 2008), consistent with visual strategies in non‐predatory, laterally eyed vertebrates that prioritize a panoramic field of view (Heesy 2004).

The data from this study provide valuable descriptive and comparative data of ocular biometry, refractive status, skull morphology, and estimated visual‐field geometry in reindeer. It highlights the need for further research into developmental processes, perceptual mechanisms, the advantages of these visual capabilities, and the potential role of sexual dimorphism in binocular vision. Overall, our findings advance the understanding of vision‐related sexual dimorphism in cervids and lay a solid foundation for future research into vision in large mammals.

## Author Contributions

**Solveig Arnegard:** data curation (equal), formal analysis (equal), investigation (equal), methodology (equal), project administration (equal), validation (equal), visualization (equal), writing – original draft (equal), writing – review and editing (equal). **Andreas Zedrosser:** conceptualization (equal), funding acquisition (equal), investigation (equal), methodology (equal), resources (equal), supervision (equal), validation (equal), writing – original draft (equal), writing – review and editing (equal). **Christine Wildsoet:** conceptualization (equal), methodology (equal), supervision (supporting), writing – review and editing (equal). **Rigmor C. Baraas:** conceptualization (equal), funding acquisition (equal), investigation (equal), methodology (equal), project administration (equal), resources (equal), supervision (equal), validation (equal), writing – original draft (equal), writing – review and editing (equal).

## Funding

This work was supported by the University of South‐Eastern Norway.

## Disclosure

Statement on Inclusion: Our study brings together authors from two countries, including scientists based in the country where the study was carried out. All authors were engaged early on with the research and study design to ensure that the diverse sets of perspectives they represent were considered from the outset. Reindeer owners were actively involved in designing the methodology, particularly regarding access to animals, handling procedures, and on‐site logistics. After the research is completed, the results will be communicated to them in an accessible format to support their own use and decision‐making.

## Conflicts of Interest

The authors declare no conflicts of interest.

## Supporting information


**Appendix S1:** Head position.
**Appendix S2:** Skull morphology.
**Appendix S3:** Linear regression models and ANOVA analysis.
**Appendix S4:** Descriptive statistics.

## Data Availability

Data available from the DataverseNO Repository: https://dataverse.no/previewurl.xhtml?token=6ca93bcd‐4ae4‐4d7c‐bc4b‐daabce894e5e.

## References

[ece374530-bib-0001] Acar, K. , L. Kiorpes , J. A. Movshon , and M. A. Smith . 2019. “Altered Functional Interactions Between Neurons in Primary Visual Cortex of Macaque Monkeys With Experimental Amblyopia.” Journal of Neurophysiology 122, no. 6: 2243–2258. 10.1152/jn.00232.2019. PMC6966320 31553685

[ece374530-bib-0002] American Academy of Ophthalmology . 2024. “Retinoscopy.” EyeWiki.

[ece374530-bib-0003] Andersson, M. 1994. Sexual Selection. Vol. 72. Princeton University Press. 10.2307/j.ctvs32s1x.

[ece374530-bib-0005] Blant, M. , and J. Gaillard . 2004. “Use of Biometric Body Variables as Indicators of Roe Deer ( *Capreolus capreolus* ) Population Density Changes.” Game & Wildlife Science 21, no. 1: 21–40.

[ece374530-bib-0007] Bowyer, R. T. 2004. “Sexual Segregation in Ruminants: Definitions, Hypotheses, and Implications for Conservation and Management.” Journal of Mammalogy 85, no. 6: 1039–1052. 10.1644/BBL-002.1.

[ece374530-bib-0008] Bracun, A. , A. D. Ellis , and C. Hall . 2014. “Retinoscopic Survey of 333 Horses and Ponies in the UK.” Veterinary Ophthalmology 17, no. s1: 90–96. 10.1111/vop.12158. 24636019

[ece374530-bib-0009] Bueno, J. M. , M. Lo Sapio , J. M. Sanes , and J. Seva . 2017. “Analysis of the Ocular Refractive State in Fighting Bulls: Astigmatism Prevalence.” BioMed Research International 2017: 1–7. 10.1155/2017/8203269. PMC5688361 29230414

[ece374530-bib-0011] Clutton‐Brock, T. H. , and A. C. J. Vincent . 1991. “Sexual Selection and the Potential Reproductive Rates of Males and Females.” Nature 351, no. 6321: 58–60. 10.1038/351058a0. 2027382

[ece374530-bib-0012] Cobo, R. , A. Navarro‐Sempere , Y. Segovia , and M. García . 2025. “Adaptations of the Vertebrate Retina to Low‐Light Conditions: A Review.” Anatomia, Histologia, Embryologia 54, no. 4: 1–12. 10.1111/ahe.70042. PMC12093289 40396371

[ece374530-bib-0013] Cronin, T. W. , S. Johnsen , N. J. Marshall , and E. J. Warrant . 2014. “Spatial Vision.” In Visual Ecology, 116–145. Princeton University Press. http://www.jstor.org/stable/j.ctt6wq1c9.

[ece374530-bib-0014] D'Angelo, G. J. , A. Glasser , M. Wendt , et al. 2008. “Visual Specialization of an Herbivore Prey Species, the White‐Tailed Deer.” Canadian Journal of Zoology 86, no. 7: 735–743. 10.1139/Z08-050.

[ece374530-bib-0015] Davidson, M. G. 1997. “Clinical Retinoscopy for the Veterinary Ophthalmologist.” Veterinary and Comparative Ophthalmology 7, no. 2: 128–137.

[ece374530-bib-0016] Dimitrova, M. , and S. Merilaita . 2009. “Prey Concealment: Visual Background Complexity and Prey Contrast Distribution.” Behavioral Ecology 21, no. 1: 176–181. 10.1093/beheco/arp174.

[ece374530-bib-0017] Direktoratet for forvaltning og økonomistyring [The Norwegian Agency for Public and Financial Management] . 2024. “Ressursregnskap for reindriftsnæringen ‐ For reindriftsåret 1. April 2023–31. Mars 2024 [Resource Accounts for the Reindeer Husbandry Industry – For the Reindeer Husbandry Year 1 April 2023–31 March 2024].” https://kudos.dfo.no/documents/290951/files/43881.pdf.

[ece374530-bib-0018] Espmark, Y. 1964. “Studies in Dominance‐Subordination Relationship in a Group of Semi‐Domestic Reindeer ( *Rangifer tarandus* L.).” Animal Behaviour 12, no. 4: 420–426. 10.1016/0003-3472(64)90061-2.

[ece374530-bib-0020] Flitcroft, D. I. 2014. “Emmetropisation and the Aetiology of Refractive Errors.” Eye 28, no. 2: 169–179. 10.1038/eye.2013.276. PMC3930278 24406411

[ece374530-bib-0021] Geist, V. 1999. Deer of the World: Their Evolution, Behaviour, and Ecology. Swan Hill Press.

[ece374530-bib-0022] Gur, M. , and J. G. Sivak . 1979. “Refractive State of the Eye of a Small Diurnal Mammal: The Ground Squirrel.” Optometry and Vision Science 56, no. 11: 689–695. 10.1002/j.2330-9512.1979.tb02729.x. 546228

[ece374530-bib-0023] Hagen, L. A. , S. J. Gilson , M. N. Akram , and R. C. Baraas . 2019. “Emmetropia Is Maintained Despite Continued Eye Growth From 16 to 18 Years of Age.” Investigative Ophthalmology & Visual Science 60, no. 13: 4178–4186. 10.1167/iovs.19-27289. 31596926

[ece374530-bib-0024] Harman, A. M. , S. Moore , R. Hoskins , and P. Keller . 1999. “Horse Vision and an Explanation for the Visual Behaviour Originally Explained by the Ramp Retina.” Equine Veterinary Journal 31, no. 5: 384–390. 10.1111/j.2042-3306.1999.tb03837.x. 10505953

[ece374530-bib-0025] Heesy, C. P. 2004. “On the Relationship Between Orbit Orientation and Binocular Visual Field Overlap in Mammals.” Anatomical Record. Part A, Discoveries in Molecular, Cellular, and Evolutionary Biology 281A, no. 1: 1104–1110. 10.1002/ar.a.20116. 15470671

[ece374530-bib-0026] Heesy, C. P. 2008. “Ecomorphology of Orbit Orientation and the Adaptive Significance of Binocular Vision in Primates and Other Mammals.” Brain, Behavior and Evolution 71: 54–67. 10.1159/000108621. 17878718

[ece374530-bib-0027] Hewitt, D. G. 2011. Biology and Management of White‐Tailed Deer. 1st ed. CRC Press. 10.1201/9781482295986.

[ece374530-bib-0028] Holand, Ø. , H. Gjøstein , A. Losvar , et al. 2004. “Social Rank in Female Reindeer ( *Rangifer tarandus* ): Effects of Body Mass, Antler Size and Age.” Journal of Zoology 263, no. 4: 365–372. 10.1017/S0952836904005382.

[ece374530-bib-0029] Holden, A. L. , and J. C. Low . 1989. “Binocular Fields With Lateral‐Eyed Vision.” Vision Research 29, no. 3: 361–367. 10.1016/0042-6989(89)90084-9. 2773346

[ece374530-bib-0030] Horth, L. 2007. “Sensory Genes and Mate Choice: Evidence That Duplications, Mutations, and Adaptive Evolution Alter Variation in Mating Cue Genes and Their Receptors.” Genomics 90, no. 2: 159–175. 10.1016/j.ygeno.2007.03.021. 17544617

[ece374530-bib-0031] Howland, H. C. , S. Merola , and J. R. Basarab . 2004. “The Allometry and Scaling of the Size of Vertebrate Eyes.” Vision Research 44, no. 17: 2043–2065. 10.1016/j.visres.2004.03.023. 15149837

[ece374530-bib-0032] Howland, H. C. , M. Rowland , and C. J. Murphy . 1993. “Refractive State of the Rhinoceros.” Vision Research 33, no. 18: 2649–2651. 10.1016/0042-6989(93)90224-K. 8296461

[ece374530-bib-0033] Howlett, M. H. C. , and S. A. McFadden . 2007. “Emmetropization and Schematic Eye Models in Developing Pigmented Guinea Pigs.” Vision Research 47, no. 9: 1178–1190. 10.1016/j.visres.2006.12.019. 17360016

[ece374530-bib-0034] Hughes, A. 1977. “The Topography of Vision in Mammals of Contrasting Life Style: Comparative Optics and Retinal Organisation.” In The Visual System in Vertebrates, edited by F. Crescitelli , 613–756. Springer Berlin Heidelberg. 10.1007/978-3-642-66468-7_11.

[ece374530-bib-0035] Isenberg, S. J. , D. Neumann , P. Y. Y. Cheong , Y. L. F. Ling , L. C. McCall , and A. J. Ziffer . 1995. “Growth of the Internal and External Eye in Term and Preterm Infants.” Ophthalmology 102, no. 5: 827–830. 10.1016/S0161-6420(95)30950-5. 7777283

[ece374530-bib-0037] Keeler . 2024. “A‐Scan Plus Connect User's Guide.” https://www.keelerglobal.com/wp‐content/uploads/2024/03/A‐Scan‐Plus‐Connect‐Rev‐D.pdf.

[ece374530-bib-0038] Land, M. F. , and D.‐E. Nilsson . 2012. Animal Eyes. 2nd ed. Oxford University Press.

[ece374530-bib-0039] Larsen, J. S. 1971. “The Sagittal Growth of the Eye. 1. Ultrasonic Measurement of the Depth of the Anterior Chamber From Birth to Puberty.” Acta Ophthalmologica 49, no. 2: 239–262. 10.1111/j.1755-3768.1971.tb00949.x. 5109787

[ece374530-bib-0040] Lincoln, G. A. 1992. “Biology of Antlers.” Journal of Zoology 226, no. 3: 517–528. 10.1111/j.1469-7998.1992.tb07495.x.

[ece374530-bib-0041] Loison, A. , and O. Strand . 2005. “Allometry and Variability of Resource Allocation to Reproduction in a Wild Reindeer Population.” Behavioral Ecology 16, no. 3: 624–633. 10.1093/beheco/ari037.

[ece374530-bib-0043] McShea, W. J. , W. H. Schlesinger , and R. S. Ostfeld . 2012. “Ecology and Management of White‐Tailed Deer in a Changing World.” Annals of the New York Academy of Sciences 1249, no. 1: 45–56. 10.1111/j.1749-6632.2011.06376.x. 22268688

[ece374530-bib-0044] Melnycky, N. A. , R. B. Weladji , Ø. Holand , and M. Nieminen . 2013. “Scaling of Antler Size in Reindeer ( *Rangifer tarandus* ): Sexual Dimorphism and Variability in Resource Allocation.” Journal of Mammalogy 94, no. 6: 1371–1379. 10.1644/12-mamm-a-282.1.

[ece374530-bib-0045] Mitchell, G. , D. G. Roberts , S. J. v. Sittert , and J. D. Skinner . 2013. “Orbit Orientation and Eye Morphometrics in Giraffes ( *Giraffa camelopardalis* ).” African Zoology 48, no. 2: 333–339. 10.3377/004.048.0211.

[ece374530-bib-0047] Morrison, D. , and P. Whitridge . 1997. “Estimating the Age and Sex of Caribou From Mandibular Measurements.” Journal of Archaeological Science 24, no. 12: 1093–1106. 10.1006/jasc.1996.0189.

[ece374530-bib-0048] Murphy, C. J. , T. J. Kern , and H. C. Howland . 1992. “Refractive State, Corneal Curvature, Accommodative Range and Ocular Anatomy of the Asian Elephant ( *Elephas maximus* ).” Vision Research 32, no. 11: 2013–2021. 10.1016/0042-6989(92)90062-N. 1304078

[ece374530-bib-0049] Newman, B. A. , and G. J. D'Angelo . 2024. “A Review of Cervidae Visual Ecology.” Animals 14, no. 3: 420. 10.3390/ani14030420. PMC10854973 38338063

[ece374530-bib-0050] Nieminen, M. , and C. J. Petersson . 1990. “Growth and Relationship of Live Weight to Body Measurements in Semi‐Domesticated Reindeer ( *Rangifer tarandus tarandus* L.).” Rangifer 10, no. 3: 353–361. 10.7557/2.10.3.879.

[ece374530-bib-0051] Nilsen, N. G. , S. J. Gilson , H. Lindgren , M. Kjærland , H. R. Pedersen , and R. C. Baraas . 2023. “Seasonal and Annual Change in Physiological Ocular Growth of 7‐ to 11‐Year‐Old Norwegian Children.” Investigative Ophthalmology & Visual Science 64, no. 15: 1–14. 10.1167/iovs.64.15.10. PMC10709800 38064230

[ece374530-bib-0052] Norton, T. T. , and N. A. McBrien . 1992. “Normal Development of Refractive State and Ocular Component Dimensions in the Tree Shrew ( *Tupaia belangeri* ).” Vision Research 32, no. 5: 833–842. 10.1016/0042-6989(92)90026-f. 1604852

[ece374530-bib-0053] Ofri, R. , S. Millodot , Y. Tadmor , et al. 2004. “The Development of the Refractive State in the Newborn Thomson Gazelle.” Journal of Comparative Physiology. A, Neuroethology, Sensory, Neural, and Behavioral Physiology 190: 831–835. 10.1007/s00359-004-0538-z. 15309483

[ece374530-bib-0054] Pape, R. , and J. Löffler . 2015. “Seasonality of Habitat Selection Shown to Buffer Alpine Reindeer Pastoralism Against Climate Variability.” Ecosphere 6, no. 12: 1–9. 10.1890/ES15-00169.1.

[ece374530-bib-0055] Pérez‐Barbería, F. , S. Ramsay , R. Hooper , et al. 2014. “The Influence of Habitat on Body Size and Tooth Wear in Scottish Red Deer ( *Cervus elaphus* ).” Canadian Journal of Zoology 93: 61–70. 10.1139/cjz-2014-0150.

[ece374530-bib-0056] Piggins, D. , and C. J. C. Phillips . 1996. “The Eye of the Domesticated Sheep With Implications for Vision.” Animal Science 62, no. 2: 301–308. 10.1017/s1357729800014612.

[ece374530-bib-0057] Qiao‐Grider, Y. , L. F. Hung , C. S. Kee , R. Ramamirtham , and E. L. Smith . 2007. “Normal Ocular Development in Young Rhesus Monkeys ( *Macaca mulatta* ).” Vision Research 47, no. 11: 1424–1444. 10.1016/j.visres.2007.01.025. PMC1995079 17416396

[ece374530-bib-0058] R Core Team . 2024. A Language and Environment for Statistical Computing. R Foundation for Statistical Computing. https://www.R‐project.org/.

[ece374530-bib-0059] Read, J. C. A. 2021. “Binocular Vision and Stereopsis Across the Animal Kingdom.” Annual Review of Vision Science 7, no. 1: 389–415. 10.1146/annurev-vision-093019-113212. 34283925

[ece374530-bib-0060] Rodriguez‐Martinez, A. , B. Zhou , M. K. Sophiea , et al. 2020. “Height and Body‐Mass Index Trajectories of School‐Aged Children and Adolescents From 1985 to 2019 in 200 Countries and Territories: A Pooled Analysis of 2181 Population‐Based Studies With 65 Million Participants.” Lancet 396, no. 10261: 1511–1524. 10.1016/S0140-6736(20)31859-6. PMC7658740 33160572

[ece374530-bib-0061] Røed, K. H. , Ø. Holand , M. E. Smith , H. Gjøstein , J. Kumpula , and M. Nieminen . 2002. “Reproductive Success in Reindeer Males in a Herd With Varying Sex Ratio.” Molecular Ecology 11, no. 7: 1239–1243. 10.1046/j.1365-294X.2002.01509.x. 12074731

[ece374530-bib-0063] Ruckstuhl, K. , and P. Neuhaus . 2000. “Sexual Segregation in Ungulates: A New Approach.” Behaviour 137, no. 3: 361–377. 10.1163/156853900502123.

[ece374530-bib-0065] Rull‐Cotrina, J. , J. M. Molleda , J. Gallardo , and E. Martín‐Suárez . 2013. “Refractive State of the Spanish Thoroughbred Horse: A Comparison With the Crossbred Horse.” Veterinary Ophthalmology 16, no. 1: 25–28. 10.1111/j.1463-5224.2012.01014.x. 22487240

[ece374530-bib-0066] Saukko, P. , and B. Knight . 2015. Knight's Forensic Pathology. 4th ed. CRC Press.

[ece374530-bib-0067] Schneider, C. A. , W. S. Rasband , and K. W. Eliceiri . 2012. “NIH Image to ImageJ: 25 Years of Image Analysis.” Nature Methods 9, no. 7: 671–675. 10.1038/nmeth.2089. PMC5554542 22930834

[ece374530-bib-0068] Sivak, J. G. , H. C. Howland , J. West , and J. Weerheim . 1989. “The Eye of the Hooded Seal, *Cystophora cristata* , in Air and Water.” Journal of Comparative Physiology. A, Sensory, Neural, and Behavioral Physiology 165, no. 6: 771–777. 10.1007/BF00610875. 2810150

[ece374530-bib-0069] Skogland, T. 1983. “The Effects of Density Dependent Resource Limitation on Size of Wild Reindeer.” Oecologia 60, no. 2: 156–168. 10.1007/BF00379517. 28310482

[ece374530-bib-0070] Srivannaboon, S. , C. Chirapapaisan , P. Nantasri , M. Chongchareon , and P. Chonpimai . 2013. “Agreement of IOL Power and Axial Length Obtained by IOLMaster 500 vs IOLMaster 500 With Sonolink Connection.” Graefes Archive for Clinical and Experimental Ophthalmology 251, no. 4: 1145–1149. 10.1007/s00417-012-2222-2. 23314479

[ece374530-bib-0071] Tideman, W. , J. R. Polling , H. Vingerling , et al. 2018. “Axial Length Growth and the Risk of Developing Myopia in European Children.” Acta Ophthalmologica 96, no. 3: 301–309. 10.1111/aos.13603. PMC6002955 29265742

[ece374530-bib-0072] Tisdale, V. , and E. Fernández‐Juricic . 2009. “Vigilance and Predator Detection Vary Between Avian Species With Different Visual Acuity and Coverage.” Behavioral Ecology 20, no. 5: 936–945. 10.1093/beheco/arp080.

[ece374530-bib-0073] Trivers, R. 1972. “Parental Investment and Sexual Selection.” In Sexual Selection and the Descent of Man, 1871–1971, edited by B. Campbell , 52–95. Aldine.

[ece374530-bib-0074] Troilo, D. , E. L. Smith , D. L. Nickla , et al. 2019. “Imi – Report on Experimental Models of Emmetropization and Myopia.” Investigative Ophthalmology & Visual Science 60, no. 3: M31–M88. 10.1167/iovs.18-25967. PMC6738517 30817827

[ece374530-bib-0075] Wallman, J. , and J. Winawer . 2004. “Homeostasis of Eye Growth and the Question of Myopia.” Neuron 74, no. 1: 447–468. 10.1016/j.neuron.2012.03.018. 15312645

[ece374530-bib-0076] Walls, G. L. 1942. The Vertebrate Eye and Its Adaptive Radiation. Cranbrook Institute of Science. https://www.biodiversitylibrary.org/item/31207.

[ece374530-bib-0077] Weladji, R. B. , A. Mysterud , Ø. Holand , and D. Lenvik . 2002. “Age‐Related Reproductive Effort in Reindeer ( *Rangifer tarandus* ): Evidence of Senescence.” Oecologia 131, no. 1: 79–82. 10.1007/s00442-001-0864-6. 28547513

[ece374530-bib-0078] Zadnik, K. , R. E. Manny , J. A. Yu , et al. 2003. “Ocular Component Data in Schoolchildren as a Function of Age and Gender.” Optometry and Vision Science 80, no. 3: 226–236. 10.1002/j.1538-9235.2003.tb01623.x. 12637834

[ece374530-bib-0079] Zuur, A. F. , E. N. Ieno , and G. M. Smith . 2007. Analysing Ecological Data. 1st ed. Springer New York. 10.1007/978-0-387-45972-1.

