# Supplementary material for: Sex, Age, and Skull Morphology Effects on Eye Size and Refractive Errors in Reindeer
Source: Ecol Evol. 2026 Nov 1;16(11):e74530. doi: 10.1002/ece3.74530 (PMC13724309; doi:10.1002/ece3.74530)
Supplement: Supplementary file 1 [file ECE3-16-e74530-s001.docx]

# Appendix S1: Head position (A)

**Figure A1.** Image of the reindeer head positioned on a custom-designed stand, oriented to simulate head posture during walking, i.e., raised head.

# Appendix S2: Skull morphology (B)

| A:   | B:   |
| --- | --- |
| C:   | D:   |

**Figure B1**. Illustration of the skull and orbital parameters measured using a digital calliper from skulls of semi-domestic reindeer collected in south-central Norway, December 2023: mandible length (A), interorbital anterior distance IOAD (B), interorbital posterior distance IOPD (C), and orbital diameter (D).

# Appendix S3: Linear regression models and ANOVA analysis (C)

Ocular dimensions

*Axial length ~ Mandible length*

There was a significant positive association between AXL and mandible length, and the final model (AXL = 16.66 + 0.045 * mandible length + (-0.781) * Sex (M), F = 96.45, p < 0.001, R^2^ = 0.74) included sex as a covariate, as it significantly improved the model fit compared with a model including mandible length only (F = 23.77, p <0.001). The interaction between mandible length and sex did not yield a significant improvement (F = 2.19, p = 0.144), but the effect of both mandible length and sex, as individual factors, was significant (t = 13.84, p < 0.001 and t = -4.83, p < 0.001, respectively), indicating that male have shorter axial length compared with females, when controlling for mandible length (Fig. 4A).

*Axial length ~ Interorbital distance*

There was a significant positive association between AXL and interorbital distance, and the final model (AXL = 15.27 + 0.092 * Interorbital distance + (-0.989) * Sex (M), F = 84.13, p < 0.001, R^2^ = 0.713) included sex as a covariate, as it significantly improved the model fit compared with a model including interorbital distance only (F = 32.81, p <0.001). The interaction between interorbital distance and sex did not yield a significant improvement (F = 2.97, p = 0.090), but the effect of both interorbital distance and sex, as individual factors, was significant (t = 12.93, p < 0.001 and t = -5.64, p < 0.001, respectively), indicating that male have shorter axial length compared with females, when controlling for interorbital distance (Fig. 4B).

*Axial length ~ Orbital diameter*

We found a significant positive association between AXL and orbital diameter, and the final model (AXL = 7.96 + 0.464 * Orbital diameter + (-0.458) * Sex (M), F = 64.13, p < 0.001, R^2^ = 0.653) included sex as a covariate, as it significantly improved the model fit compared with a model including orbital diameter only (F = 6.30, p = 0.015). The interaction between orbital diameter and sex did not yield a significant improvement (F = 0.37, p = 0.544), but the effect of both orbital diameter and sex was significant (t = 4.75, p < 0.001 and t = -2.52, p < 0.014, respectively), indicating that male have shorter axial length compared with females, when controlling for orbital diameter (Fig. 4C).

Refractive errors

*SER ~ Axial length*

Spherical equivalent refraction (SER) was analysed as the response variable, with axial length (AXL) as the primary predictor. There was a significant negative association between SER and AXL (SER = 4.429 + (-0.133) * AXL, F = 9.579, p = 0.003), indicating that SER decreases with increasing axial length for both sexes, and the model explained 11.8% of the variance in axial length (R² = 0.118). The effect of sex was not significant (p = 0.099), nor was the interaction between SER and sex (p = 0.777).

Anterior blind zone

*Anterior blind zone ~ Mandible length*

There was a significant positive association between anterior blind zones and mandible length, and the final model (Anterior blind zone = -39.186 + 1.20 * mandible length, F = 29.45, p < 0.001, R^2^ = 0.295) included mandible length as the sole predictor. The effect of sex was not statistically significant (F = 0.911, p = 0.3435), nor was the interaction between mandible length and sex (F = 0.000, p = 0.997) (Fig. 6B)

**Table C1.** Summary of hierarchical linear regression models for each predictor pair to explore the relationships between different parameters and sex-related differences, in semi-domestic reindeer collected in south-central Norway, December 2023. The table includes predictor variables, regression estimates (*B* ± SE, β), t-values with corresponding p-values, adjusted R² values, F-statistics with associated p-values, and significance from ANOVA model comparisons (ANOVA (p)). All hierarchical linear regression models start with an intercept-only null model (Model 0) with the formulation y~1.

| **Predictor pair** | **Model #** | **Predictor variables** | **Estimates (*B* ± SE, β)** | **t-value, p-value** | **Adj. R²** | **F-statistics (p)** | **ANOVA (p)** |
| --- | --- | --- | --- | --- | --- | --- | --- |
| Axial length ~ Mandible length | 0 | Intercept | 26.710 ± 0.150 | 178.7, <0.001 |  |  |  |
|  | 1 | Intercept | 17.178 ± 0.851 | 20.18, <0.001 |  |  |  |
|  |  | Mandible length | 0.041 ± 0.004, 0.811 | 11.26, <0.001 | 0.652 | 126.7, <0.001 | <0.001 |
|  | 2 | Intercept | 16.661± 0.744 | 22.404, <0.001 |  |  |  |
|  |  | Mandible length + sex | -0.781 ± 0.162, -0.312 | -4.831, <0.001 | 0.740 | 96.45, <0.001 | <0.001 |
|  | 3 | Intercept | 15.405 ± 1.123 | 13.712, <0.001 |  |  |  |
|  |  | Mandible length * sex | -0.010 ± 0.007, -0.935 | -1.481, 0.143 | 0.745 | 66.21, <0.001 | 0.144 |
| Axial length ~ Interorbital distance | 0 | Intercept | 26.710 ± 0.150 | 178.7, <0.001 |  |  |  |
|  | 1 | Intercept | 16.560 ± 1.057 | 15.665, <0.001 |  |  |  |
|  |  | Interorbital distance | 0.078 ± 0.008, 0.765 | 9.641, <0.001 | 0.579 | 92.95, <0.001 | <0.001 |
|  | 2 | Intercept | 15.279 ± 0.902 | 16.944, <0.001 |  |  |  |
|  |  | Interorbital distance + sex | -0.989 ± 0.175, -0.396 | -5.644, <0.001 | 0.713 | 84.13, <0.001 | <0.001 |
|  | 3 | Intercept | 13.372 ± 1.420 | 9.419, <0.001 |  |  |  |
|  |  | Interorbital distance * sex | -0.025 ± 0.014, -1.343 | -1.722, 0.090 | 0.721 | 58.77, <0.001 | 0.090 |
| Axial length ~ Orbital diameter | 0 | Intercept | 26.710 ± 0.150 | 178.7, <0.001 |  |  |  |
|  | 1 | Intercept | 8.262 ± 1.740 | 4.749, <0.001 |  |  |  |
|  |  | Orbital diameter | 0.450 ± 0.042, 0.794 | 10.618, <0.001 | 0.625 | 112.7, <0.001 | <0.001 |
|  | 2 | Intercept | 7.959 ± 1.678 | 4.745, <0.001 |  |  |  |
|  |  | Orbital diameter + sex | -0.458 ± 0.182, -0.183 | -2.522, 0.014 | 0.653 | 64.13, <0.001 | 0.015 |
|  | 3 | Intercept | 6.772 ± 2.573 | 2.632, 0.011 |  |  |  |
|  |  | Orbital diameter * sex | -0.051 ± 0.084, -0.844 | -0.611, 0.544 | 0.650 | 42.46, <0.001 | 0.544 |
| SER ~ Axial length | 0 | Intercept | 0.871 ± 0.056 | 15.44, <0.001 |  |  |  |
|  | 1 | Intercept | 4.429 ± 1.151 | 3.849, <0.001 |  |  |  |
|  |  | Axial length | -0.133 ± 0.043, -0.363 | -3.095, 0.003 | 0.118 | 9.579, 0.003 | 0.003 |
|  | 2 | Intercept | 4.199 ± 1.143 | 3.674, <0.001 |  |  |  |
|  |  | Axial length + sex | 0.178 ± 0.106, 0.194 | 1.676, 0.099 | 0.143 | 6.332, 0.003 | 0.101 |
|  | 3 | Intercept | 3.889 ± 1.590 | 2.446, 0.017 |  |  |  |
|  |  | Axial length * sex | -0.024 ± 0.086, -0.710 | -0.284, 0.777 | 0.130 | 4.186, 0.009 | 0.777 |
| Anterior blind zone ~ Mandible length | 0 | Intercept | 242.76 ± 6.55 | 37.06, <0.001 |  |  |  |
|  | 1 | Intercept | -39.186 ± 52.243 | -0.750, 0.456 |  |  |  |
|  |  | Mandible length | 1.201± 0.221, 0.553 | 5.427, <0.001 | 0.295 | 29.45, <0.001 | <0.001 |
|  | 2 | Intercept | -47.045 ± 52.908 | -0.889, 0.377 |  |  |  |
|  |  | Mandible length + sex | -11.306 ± 11.757, -0.102 | -0.962, 0.340 | 0.294 | 15.17, <0.001 | 0.344 |
|  | 3 | Intercept | -46.790 ± 83.133 | -0.563, 0.575 |  |  |  |
|  |  | Mandible length * sex | 0.002 ± 0.474, 0.004 | 0.004, 0.997 | 0.283 | 9.961, <0.001 | 0.997 |

# Appendix S4: Descriptive statistics (D)

**Table D1.** Descriptive statistics (mean, median and range) for axial length (AXL), mandible length, interorbital anterior distance (IOAD), interorbital posterior distance (IOPD), and orbital diameter (Orb. ⌀), grouped according to developmental stage (calves and adults), of semi-domestic reindeer (female and male) collected in South-Central Norway, December 2023 (n=69).

|  | CALVES | | | |  | ADULTS | | | |
| --- | --- | --- | --- | --- | --- | --- | --- | --- | --- |
|  | n | Mean | Median | Range |  | n | Mean | Median | Range |
| AXL right eye (mm) |  |  |  |  |  |  |  |  |  |
| All | 35 | 25.9 | 25.8 | [25.0 – 27.0] |  | 34 | 27.9 | 27.7 | [26.4 – 29.7] |
| F | 17 | 25.7 | 25.7 | [25.0 – 26.4] |  | 10 | 28.6 | 28.9 | [27.2 – 29.7] |
| M | 18 | 26 | 25.9 | [25.2 – 27.0] |  | 24 | 27.6 | 27.6 | [26.4 – 28.7] |
| AXL left eye  (mm) |  |  |  |  |  |  |  |  |  |
| All | 35 | 25.7 | 25.8 | [24.2 – 27.5] |  | 34 | 27.7 | 27.8 | [25.5 – 29.4] |
| F | 17 | 25.9 | 25.9 | [24.3 – 27.5] |  | 10 | 28.4 | 28.4 | [27.8 – 29.4] |
| M | 18 | 25.6 | 25.7 | [24.2 – 26.8] |  | 24 | 27.4 | 27.5 | [25.5 – 28.6] |
| Mandible length (mm) |  |  |  |  |  |  |  |  |  |
| All | 35 | 212.2 | 212.9 | [191.7 – 235.0] |  | 34 | 258.0 | 255.7 | [236.6 – 283.1] |
| F | 17 | 208.2 | 209.1 | [191.7 – 219.9] |  | 10 | 256.2 | 255.5 | [244.4 – 269.3] |
| M | 18 | 216.0 | 215.8 | [199.7 – 235.0] |  | 24 | 258.7 | 255.7 | [236.6 – 283.1] |
| IOAD (mm) |  |  |  |  |  |  |  |  |  |
| All | 35 | 108.8 | 109.1 | [97.7 – 118] |  | 34 | 130.8 | 130.7 | [120.5 – 143.5] |
| F | 17 | 106.6 | 106.8 | [97.7 – 114.1] |  | 10 | 127.7 | 127.7 | [120.5 – 134.7] |
| M | 18 | 110.9 | 110.9 | [103.3 – 118] |  | 24 | 132.1 | 130.9 | [124.6 – 143.5] |
| IOPD (mm) |  |  |  |  |  |  |  |  |  |
| All | 35 | 129.8 | 129.2 | [122.2 – 138.5] |  | 34 | 152.6 | 152.4 | [143.6 – 166.8] |
| F | 17 | 126.9 | 127.0 | [122.2 – 133.0] |  | 14 | 149.2 | 148.7 | [143.6 – 160.6] |
| M | 18 | 132.6 | 132.3 | [124.0 – 138.5] |  | 24 | 154.0 | 153.6 | [145.6 – 166.8] |
| Orb. ⌀ right eye (mm) |  |  |  |  |  |  |  |  |  |
| All | 35 | 39.0 | 39.1 | [36.9 – 40.9] |  | 34 | 42.7 | 42.7 | [39.5 – 46.1] |
| F | 17 | 38.9 | 39.1 | [36.9 – 40.3] |  | 10 | 42.8 | 43.0 | [39.5 – 44.8] |
| M | 18 | 39.2 | 39.0 | [37.6 – 40.9] |  | 24 | 42.6 | 42.3 | [40.5 – 46.1] |
| Orb. ⌀ left eye (mm) |  |  |  |  |  |  |  |  |  |
| All | 35 | 39.3 | 39.3 | [37.6 – 40.6] |  | 34 | 42.9 | 42.7 | [38.7 – 46.7] |
| F | 17 | 39.2 | 39.3 | [38.0 – 40.4] |  | 10 | 43.1 | 43.3 | [38.7 – 45.1] |
| M | 18 | 39.3 | 39.2 | [37.6 – 40.6] |  | 24 | 42.8 | 42.5 | [41.0 – 46.7] |
